# Statins use and female lung cancer risk in Taiwan

**DOI:** 10.3402/ljm.v7i0.20123

**Published:** 2012-12-27

**Authors:** Shih-Wei Lai, Kuan-Fu Liao, Cheng-Li Lin, Fung-Chang Sung, Ya-Hsin Cheng

**Affiliations:** 1 School of Medicine, China Medical University, Taichung, Taiwan; 2 Department of Family Medicine, China Medical University Hospital, Taichung, Taiwan; 3 Graduate Institute of Integrated Medicine, China Medical University, Taichung, Taiwan; 4 Department of Internal Medicine, Taichung Tzu Chi General Hospital, Taichung, Taiwan; 5 Department of Health Care Administration, Central Taiwan University of Science and Technology, Taichung, Taiwan; 6 Department of Public Health, China Medical University, Taichung, Taiwan; 7 Management Office for Health Data, China Medical University Hospital, Taichung, Taiwan; 8 Department of Physiology, School of Medicine, China Medical University, Taichung, Taiwan

**Keywords:** statins, lung cancer

## Abstract

In this present study, we found that the use of rosuvastatin with cumulative using duration >12 months could correlate with 2.8-fold increased risk of lung cancer in women. We did not have specific comments on these results. Further prospective clinical studies of statins use are needed to elucidate this issue.

## Introduction

In order to clarify the association between statins use and female lung cancer risk, we extended the study period and collected more female lung cancer cases by analyzing the Taiwan National Health Insurance database from 2000 to 2010.

## Methods

There were 1117 female subjects with newly diagnosed lung cancer (based on ICD-9 codes 162.X and A-code A101), who were aged 20 years or older at the date of diagnosing lung cancer (mean age 66.5 years and standard deviation 13.4 years). In addition, 4468 control subjects without lung cancer were matched with age and index date (mean age 65.9 years and standard deviation 13.6 years). The insurance program details can be found in previously published studies (1–3). Six commercially available statins in Taiwan were analyzed, including simvastatin, fluvastatin, lovastatin, atorvastatin, pravastatin, and rosuvastatin.

## Results

The lung cancer cases were more likely to have pulmonary tuberculosis (3.58% vs. 0.92%) and chronic obstructive pulmonary disease (31.8% vs. 19.0%) (*P*<0.0001). Moreover, there were 212 subjects with statins use among lung cancer cases (19.0%) and 752 subjects with statins use among control subjects (16.8%) (*P*=0.09). There was no statistical difference in using duration of statins between lung cancer cases and control subjects (mean±SD, months, 23.40±52.86 vs. 18.85±33.64, *P*=0.13) (Table 1).

**Table 1:** Baseline characteristics between lung cancer cases and control subjects in women

	Lung cancer				
	
	No		Yes		
		
	*N=*4468		*N=*1117		
		
	*N*	%	*N*	%	*P*
Age group (years)					
20–39	123	2.8	28	2.51	0.60
40–64	1672	37.4	403	36.08	
≥ 65	2673	59.8	686	61.41	
Age (mean and SD, years)*	65.9	13.6	66.5	13.4	0.21
Co-morbidities prior to index date†					
Obesity	23	0.51	11	0.98	0.07
Pulmonary tuberculosis	41	0.92	40	3.58	<0.0001
Chronic obstructive pulmonary disease	849	19.0	355	31.8	<0.0001
Pneumoconiosis**	7	0.16	4	0.36	0.17
Tobacco use	2	0.04	0	0.00	–
Use of medications					
Statins	752	16.8	212	19.0	0.09
Using duration of statins (months, mean±SD)*	18.85	33.64	23.40	52.86	0.13
Non-statin lipid-lowering drugs	538	12.0	146	13.1	0.35

** Chi-square, Fisher's exact test

* *t*-test comparing women with and without lung cancer.

† The co-morbidities potentially associated with lung cancer were diagnosed as follows: obesity (ICD-9 codes 278.00 and 278.01, and A-code A183), pulmonary tuberculosis (ICD-9 codes 010.X, 011.X, 012.X, and 018.X), chronic obstructive pulmonary disease (ICD-9 codes 491.X, 492.X, 493.X, and 496.X), pneumoconiosis (ICD-9 codes 500,502,503, 504, and 505), and tobacco use (ICD-9 codes 305.1).

After controlling for co-variables, multiple logistic regression analysis showed that no association was detected between statins use and lung cancer risk (odds ratio=1.07, 95% CI=0.90–1.27) (Table 2). In further analysis, only use of rosuvastatin with cumulative using duration > 12 months could correlate with increased risk of lung cancer (odds ratio=2.79, 95% CI=1.37–5.66), as compared with non-use of statins (Table not shown).

**Table 2:** Odds ratios and 95% confidence intervals of lung cancer associated with statins use and covariates in women

	Crude		Adjusted†	
		
Variable	OR	(95% CI)	OR	(95% CI)
Age (per one year)	1.00	(0.998, 1.01)	–	–
Co-morbidities prior to index date (yes vs. no)				
Obesity	1.92	(0.93, 3.96)	–	–
Pulmonary tuberculosis	4.01	(2.58, 6.23)	3.22	(2.06, 5.05)
Chronic obstructive pulmonary disease	1.99	(1.72, 2.30)	1.92	(1.65, 2.24)
Pneumoconiosis	2.29	(0.67, 7.84)	–	–
Medications (use vs. non-use)				
Statins	1.16	(0.98, 1.37)	1.07	(0.90, 1.27)
Non-statin lipid-lowering drugs	1.10	(0.90, 1.34)	–	–

† Adjusted for pulmonary tuberculosis and chronic obstructive pulmonary disease.

## Discussion

To date, controversy exists regarding the association between statins use and lung cancer risk. A case-control study by Khurana and colleagues in the United States showed that statins use for more than 6 months could correlate with a risk reduction of lung cancer (odds ratio=0.45, 95% CI=0.42–0.48) (4), which was contrary to Cheng and colleagues’ findings in Taiwan (odds ratio=0.82, 95% CI=0.58–1.15) (5). In this present study, we found that the use of rosuvastatin with cumulative using duration > 12 months could correlate with 2.8-fold increased risk of lung cancer in women. We did not have specific comments on these results. In our view, because of inconclusive clinical data, further prospective clinical studies of statins use are needed to clearly elucidate this issue.
